# The diverse roles of host membranes in plant–microbe interactions

**DOI:** 10.1371/journal.ppat.1013921

**Published:** 2026-02-05

**Authors:** Marie-Dominique Jolivet, Hua Wei, Isabella Gantner, Andreas Klingl, Caroline Gutjahr, Julien Gronnier

**Affiliations:** 1 Plant cell Biology, TUM School of Life Sciences, Technical University of Munich, Freising, Germany; 2 Center for Plant Molecular Biology (ZMBP), University of Tübingen, Tübingen, Germany; 3 Plant Development and Electron Microscopy, Department of Biology I, Biocenter LMU Munich, Planegg-Martinsried, Germany; 4 Max-Planck-Institute of Molecular Plant Physiology, Potsdam Science Park, Potsdam-Golm, Germany; University of Tübingen: Eberhard Karls Universitat Tubingen, GERMANY

## Introduction

Plants encounter a myriad of microorganisms that can invade with detrimental or beneficial outcomes. Taking a membrane-centric point of view, we discuss the cellular events underlying microbe-induced cell signaling, the navigation of microbes within plant tissues, and the molecular exchanges occurring at plant–microbe interfaces. We discuss the implications of individual membrane lipids and the emerging role of membrane biophysics in non-self and modified-self sensing. We highlight the common themes underlying the active role of membranes during plant interactions with viruses, bacteria, oomycetes, and fungi, and define exciting directions for future research.

## Cell membranes host microbe-induced signaling events

**Fig 1:**
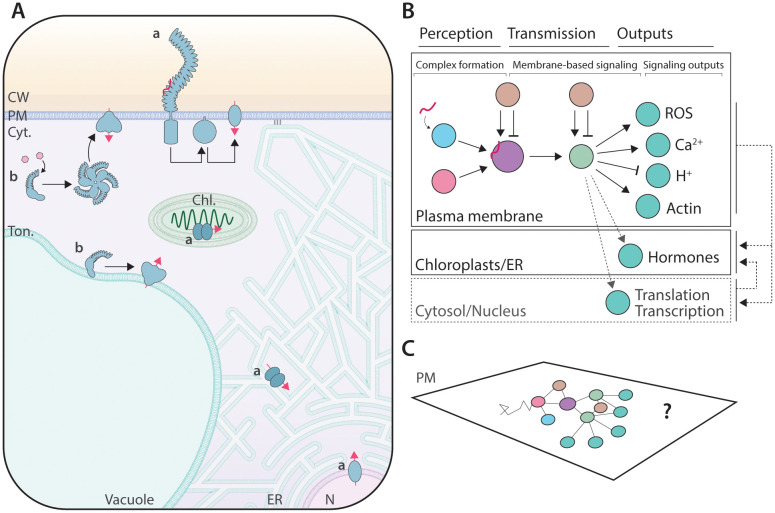
Membranes support microbe-induced cell signaling. **A**. Diverse membrane compartments support microbe-induced signaling in plant cells. Cell surface signaling involves the perception of MAMPs by plasma membrane-located receptors, which induce plasma membrane-based transduction cascades (a), leading, among other outputs, to ion fluxes at the plasma membrane and/or the nucleus and hormone production by ER and chloroplast-localized membrane proteins. Intracellular immune signaling involves direct and indirect sensing of microbial proteins by NLRs, leading to their oligomerization and the formation of tonoplast- or PM-located ion-permeable channels (b). CW: cell wall; PM: plasma membrane; Cyt.: cytosol; Ton.: tonoplast; ER: endoplasmic reticulum; N.: nucleus. **B**. Schematic representation of the subcellular distribution of microbe-induced cell signaling, which, from complex formation to the generation of signaling outputs, predominantly involves membrane proteins. **C**. Conceptual representation of the unknown spatial and temporal regulation of signaling taking place at the plasma membrane, using as an example the processes depicted in panel B.

Plants recognize microbial molecules to establish appropriate symbiotic or immune responses. A first layer of recognition is mediated by the perception of microbe-associated molecular patterns (MAMPs) by cell-surface receptors such as receptor-like kinases (RLKs) and receptor-like proteins (RLPs) at the plasma membrane. MAMP perception triggers an array of signaling events, most of which are hosted by membranes (Fig 1). Prominent examples are the perception of lipochitooligosaccharides by the LysM-RLKs NOD-FACTOR RECEPTOR KINASE 1 (NFR1) and 5 (NFR5), regulating nodule symbiosis in legumes [1,2], and the perception of the flagellin epitope flg22 by the *Arabidopsis thaliana* (hereafter Arabidopsis) FLAGELIN SENSING 2 (FLS2) [3]. Flg22-triggered signaling is initiated by the formation of a ligand-induced complex between FLS2 and its main co-receptor BRI1-ASSOCIATED RECEPTOR KINASE 1 (BAK1), which activates numerous protein kinases such as receptor-like cytoplasmic kinases (RLCKs), calcium-dependent protein kinases (CPKs), and mitogen-associated protein (MAP) kinases (MAPKs) [4]. These kinases regulate the activity of proteins generating signaling outputs, such as the production of reactive oxygen species (ROS) [5], ion fluxes [6,7], the reorganization of the cytoskeleton [8], plasma membrane-to-chloroplast signaling [9], changes in hormonal dosage [10], and transcriptional reprogramming [11]. Microbial-induced signaling also modifies symplastic communication through the modulation of plasmodesmata (PD) aperture, membrane-lined nanopores that link neighboring cells [12]. Membrane contact sites (MCSs) between the endoplasmic reticulum (ER) and the plasma membrane are prominent regulators of PD function [13] and are supported by protein tethers such as SYNAPTOTAGMIN1, which accumulates at PD upon pathogenic elicitor treatment to reduce cell-to-cell connectivity [14]. The perception of symbiotic patterns follows similar signaling principles and involves, in addition, calcium signaling from the nuclear envelope [15].

Such signaling cascades are further tightly regulated by plasma membrane-localized proteins (Fig 1B). For instance, flg22-induced FLS2-BAK1 complex formation and activation is regulated by accessory receptor kinases [16,17], protein phosphatases [18], and proteins mediating SUMOylation [19], S-acylation [20], or ubiquitination [21,22] of FLS2. Similarly, the activity and protein stability of the RLCK BOTRITYS-INDUCED KINASE 1 (BIK1) is controlled by numerous plasma membrane-associated positive and negative regulators [23–26]. Biochemical and live-cell imaging experiments have described the direct and sometimes dynamic association of these elements upon ligand perception. For instance, BIK1 is released from receptor complexes to associate with and activate downstream molecular actors [7,27]. We remain, however, largely ignorant regarding the spatial and temporal regulation of these molecular events within membranes. Several studies highlight that numerous immune and symbiotic signaling components are spatially organized into membrane nanodomains [28], such as FLS2 [29,30], RBOHD [31], and LYK3 [32], while BIK1 is dynamically stabilized within the plasma membrane upon flg22 perception [33]. Defects in plasma membrane organization have been shown to correlate with defects in cellular responses [34–37], and several case studies show that microbial proteins and metabolites affect the organization of the plasma membrane [38,39]. Upon plantago asiatica mosaic virus infection, the Arabidopsis CPK3 is recruited to plasma membrane nanodomains, phosphorylates group 1 REMORIN proteins, and limits viral propagation [37]. Despite accumulating reports linking membrane dynamics and signaling, a mechanistic understanding of how membranes regulate signaling events is often lacking.

Membranes also support intracellular immune receptor signaling. Indeed, direct and indirect perception of microbial effectors by intracellular nucleotide-binding leucine-rich repeat (NLR) receptors triggers their oligomerization into membrane-localized resistosomes that act as calcium-permeable channels for effector-triggered immunity (ETI) signaling [40,41]. Activated NLRs from different plants can target distinct membranes, including the plasma membrane and organelle membranes [42–45]. For instance, upon activation, the Arabidopsis coiled-coil (CC)–NLR HOPZ-ACTIVATED RESISTANCE 1 forms a wheel-like pentamer [46] that relocates from the cytosol to the plasma membrane [47]. Live-cell analyses of the plasma membrane recruitment of the *Nicotiana benthamiana* helper NLR Required for Cell Death 2 (NRC2) and NRC4 suggest that NRC resistosomes are organized into specialized plasma membrane environments [42,48]. Further, NRC4, but not NRC2, is dynamically recruited to the extra-haustorial membrane (EHM) during *Phytophthora infestans* [43]. The Arabidopsis helper NLR immune receptor Atypical Disease Resistance 1 (ADR1) localizes at the plasma membrane in a phospholipid-dependent manner in its resting and active states [49]. The Arabidopsis CC NLR, potentially membrane-localized 5, localizes at the Golgi and the tonoplast and initiates immune signaling at the vacuole through a yet undescribed mechanism that may deviate from canonical pore-forming resistosomes [44]. What determines the specific targeting of an intracellular immune receptor to a given membrane and to potential specialized membrane nano-environments, and how such targeting may contribute to its signaling function, is unknown. In immune signaling, the activity of cell surface and intracellular receptors mutually potentiate each other [50]. In the case of the perception of nlp20, an immunogenic epitope derived from Necrosis and Ethylene-inducing Peptide 1 (Nep1)-like proteins (NLPs), by the cell surface receptor-like protein RLP23, the co-receptor SUPPRESSOR OF BIR1-1 has been shown to physically link RLP23 with the intracellular ETI signaling module EDS1-PAD4-ADR1, thereby suggesting the formation of plasma membrane nano-environments that may contribute to ETI-PTI synergy [51].

In turn, microbial effector proteins and metabolites target plasma membrane-associated processes to promote disease [52,53]. For instance, the type 3 effector from *Xanthomonas campestris campestris* (Xcc) XopR forms membrane-associated molecular condensates that hijack actin cytoskeleton remodeling components [54] and fluidifies condensates formed by the immune RPM1-interacting protein 4 [55]. Diffusible signal factor, a quorum-sensing lipid produced by Xcc, affects plasma membrane nanodomain organization of FLS2 [39] and of the actin-nucleating protein Formin 6 [38]. AVRcap1, an effector from *Phytophthora infestans*, blocks the stepwise assembly of the tomato helper NLR SlNRC3 resistosomes [56] and its association with the *Nicotiana benthamiana* TOL9a protein, a putative member of the host ESCRT pathway, contributes to the suppression of NRC2-mediated cell death [57,58]. Another prominent example is the subversion of the plasma membrane-to-chloroplast immune signaling pathway by viral proteins and bacterial effectors [9].

## Reshaping of host membrane systems during microbial invasion

**Fig 2:**
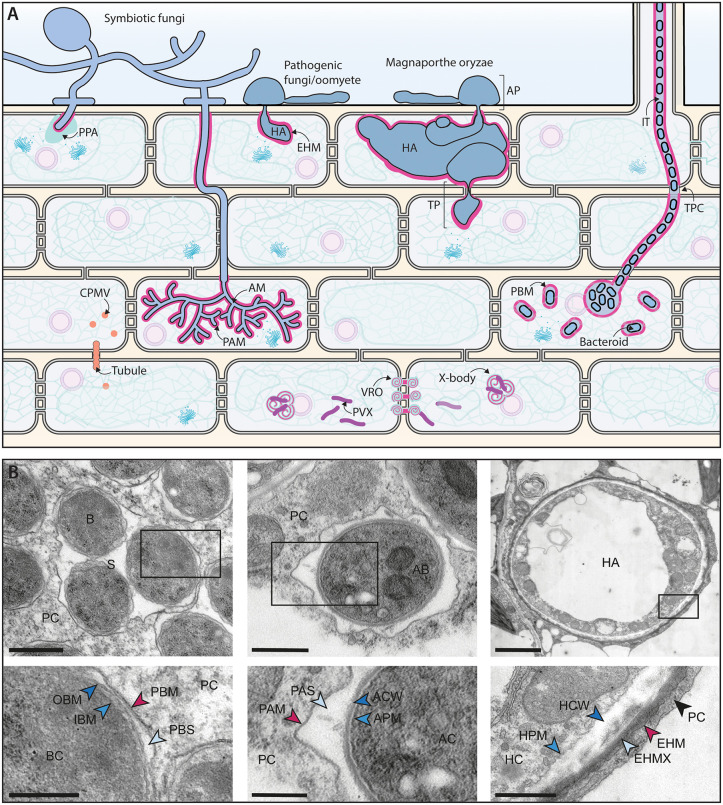
Establishment of specialized host membranes in plant-microbe interactions. **A**. Specialized plant-derived membranes are established upon microbial colonization of plant tissues. The ER-membrane-enriched pre-penetration apparatus (**PPA**) peri-fungal plant membrane delineates penetration sites of symbiotic fungi, while the plasma membrane-derived peri-arbuscular membrane (**PAM**) surrounds the arbuscule. Similarly, the extra-haustorial membrane (**EHM**) surrounds the haustoria of pathogenic fungi and oomycetes. Using the transpressorium (**TP**), a structure analogous to the appressorium (**AP**), *Magnaporthe oryzae* navigates from cell to cell through plasmodesmata (**PD**) environments. The infection thread (**IT**), a membrane invagination of the root hair cells, guides rhizobia to the root cortex. Cell-to-cell IT progression is ensured by intercellular compartments named transcellular passage clefts (**TPCs**). In the cortex, bacteroids are released into symbiosomes, intracellular compartments surrounded by the host-derived peri-bacteroid membrane (**PBM**). Potato virus X (**PVX**) hijacks ER membranes to establish viral replication organelles (**VROs**) near PD. At later stages of infection, PVX induces large ER- and Golgi-derived compartments, termed X-bodies, at the periphery of the nucleus. The cowpea mosaic virus (**CPMV**) navigates from cell to cell via the formation of tubules inside PD, and tubule formation depends on interactions between its movement protein and PD-localized proteins. Microbes are depicted in dark blue and peri-microbial plant membranes in magenta. **B**. TEM micrographs of intracellularly accommodated microbes encapsulated in plant host membranes. Left: TEM image of a symbiosome in *Lotus japonicus* containing *Mesorhizobium loti*, surrounded by plant cytoplasm (**PC**) (scale bar: 500 nm). The interface close-up shows the bacteroid cytoplasm (**BC**) enclosed by the inner/outer bacteroid membranes (**IBM/OBM**), peribacteroid space (**PBS**), and PBM (scale bar: 250 nm). Center: TEM image of a fine arbuscule branch (**AB**) in a *L. japonicus* cell with *Rhizophagus irregularis*, surrounded by **PC** (scale bar: 1 µm). The close-up shows the arbuscule cytoplasm (**AC**) enclosed by its plasma membrane (**APM**), cell wall (**ACW**), the peri-arbuscular space (**PAS**), and PAM (scale bar: 500 nm). Right: TEM image of a haustorium (**HA**) in *A. thaliana* infected with *Hyaloperonospora arabidopsidis*, surrounded by **PC** (scale bar: 2 µm). The close-up shows the haustorium cytoplasm (**HC**) enclosed by its plasma membrane (**HPM**), cell wall (**HCW**), extra-haustorial matrix (**EHMX**), and EHM (scale bar: 500 nm).

Microbial encounters induce extensive modifications of host membrane systems (Fig 2). These changes facilitate microbial navigation within plant tissues, the formation of specialized interfaces for molecular exchange, and the regulation of plant immune responses. To guide the entry of beneficial fungi and rhizobia, plants form novel membrane compartments, originating respectively from the ER-enriched pre-penetration apparatus and pre-infection thread, followed by the formation of plasma membrane-lined tube-like structures, the peri-fungal membrane, and the infection thread [59,60]. Microbes must then navigate within plant tissues, a process that requires structural adaptations. For example, during colonization by rhizobia, the extracellular matrix is remodeled to form a novel membrane-lined compartment termed the transcellular passage cleft [61]. After successful penetration, symbiotic membrane interfaces that enable controlled molecular exchanges are created. These interfaces include the peri-arbuscular membranes that surround arbuscules of arbuscular mycorrhizal fungi in root cortex cells, or peribacteroid membranes around rhizobial bacteroids to form symbiosomes [62,63]. Like symbionts, pathogenic oomycetes and fungi induce specialized membrane compartments, such as the EHM, for effector delivery to the host and nutrient uptake. The penetration of *Magnaporthe oryzae* in rice leaf epidermal cells is also accompanied by the formation of a plant-derived, membrane-rich cap termed the biotrophic interfacial complex (BIC) [64]. The BIC is notably utilized by the fungus for effector delivery [65–68]. *Phytophthora capsici* infection generates similar membranous structures at the EHM neck, which may serve as a functional analogue of the BIC [69]. For both symbionts and pathogens, the establishment of host membrane interfaces represents a remarkable increase in the plant membrane surface area, which requires considerable membrane biosynthesis. How membrane biogenesis is regulated in these contexts remains unknown and is likely defined by a complex molecular dialogue between plants and microbes.

To oppose colonization by pathogenic microbes, the plant secretory machinery delivers defense proteins and antimicrobial compounds at the site of infection [70]. MCSs allow vesicle-free exchanges of components between cell compartments and may support focal immune responses, exemplified by the local accumulation of callose mediated by proteins localized at chloroplasts-EHM MCSs [71]. Microbes, on the other hand, modulate membrane trafficking pathways [53,72], such as exocytosis [73], endocytosis [68,74–76], and autophagy [77–79], to manipulate the plant immune system, gain resources, and promote the establishment of a favorable environment [80]. Similarly, several host trafficking components are crucial for the establishment of symbiosis [81].

Akin to prokaryotic and eukaryotic invading microbes, plant viruses manipulate host membranes, inducing drastic ultrastructural modifications [72,82]. This remodeling supports viral movement and the formation of viral replication organelles (VROs) by RNA viruses. Examples of VROs include the ER-derived X-bodies generated by the potato virus X [83] and peroxisomal membrane-derived vesicle-like invaginations formed by the tomato bushy stunt virus [84]. To move from cell to cell, viruses employ various strategies to hijack PD [85]. These include the manipulation of MCSs [86], ER constriction [87], membrane-based synthesis of cell wall polymers [12], or the assembly of viral tubular structures within PD [82,88]. Viruses are not the sole organisms manipulating PD environments to navigate within plant tissues. Indeed, the fungus *Magnaporthe oryzae* forms transpressoria, specialized hyphal structures that constrict through cell regions presenting a high density of PD to colonize neighboring cells [64].

Another seemingly common theme of host colonization is the formation of extracellular membrane compartments. Host invasion by symbiotic and pathogenic fungi has been shown to coincide with the formation of extracellular vesicles (EVs), and membrane tubules are suspected to mediate inter-organismic signals and nutrient exchanges [89,90]. The fusion of plant multivesicular bodies (MVBs) with the plasma membrane is seen as a likely basis for the release of EVs in the apoplast [89]. EVs carry small RNA mediating RNA interference in pathogenic interactions [91], and a similar bidirectional cross-kingdom RNA interference is thought to occur in mutualistic symbioses [92]. EVs have also been proposed to be leveraged by Turnip mosaic virus (TuMV) to navigate in the apoplast via the fusion of MVBs with the plasma membrane at PD [93].

## The emerging role of membrane biophysics

Cell membranes display defined membrane biophysical properties, including thickness, order, curvature, and permeability, which contribute to organelle identity [94,95]. For instance, the bilayer of the plasma membrane is far thicker and more rigid than that of the ER membrane [96]. Such distinctive parameters are thought to contribute to organelle function. Accumulating evidence indicates that the regulation and the sensing of membrane biophysics regulate the outcome of plant–microbe interactions. For instance, surfactin, a lipopeptide produced by *Bacillus* spp, inserts into the plasma membrane by binding to glucosylceramides, outer-leaflet sphingolipids, and induces increased membrane lipid packing and curvature [97]. Such changes in membrane properties are thought to activate mechanosensitive channels and to initiate an unconventional immune signaling pathway, leading to systemic acquired resistance [97]. These observations add to previously established links between mechanosensitive channels and immunity. For instance, gain-of-function mutants for the mechanosensitive channels MSL10 promote resistance against bacteria and induce autoimmune phenotypes [98,99], while phosphorylation of the mechanosensitive channels OSCA1.3 and OSCA1.7 mediates stomatal immunity [7,100]. Surfactin is not the sole microbial-derived metabolite linking membrane biophysics and plant immunity. Indeed, bacterial rhamnolipids have been shown to increase the fluidity of model membranes, to induce the production of ROS, and to promote plant immunity [101,102]. The effect of rhamnolipids relies on sphingolipid composition but not on tested Mid1-Complementing Activity and MSL mechanosensitive channels [101]. Likewise, fungal ergosterols have been long thought to interfere with the properties of plant membranes and shown to induce immune responses [103,104]. Nonetheless, the implication of protein cell surface receptors in rhamnolipids or ergosterol-triggered signaling cannot be excluded [101,104]. Outer membrane vesicles from *Xanthomonas campestris* have been proposed to fuse with the plant plasma membrane and to increase membrane order, an effect which is associated with an increase in disease resistance [105]. These studies suggest that plants sense changes in membrane biophysics and consequently mount immune responses.

Changes in membrane properties have also been observed upon perception of MAMPs. Indeed, the perception of flg22 or oligosaccharides leads to an increase in membrane order that occurs in a NADPH-dependent manner [106]. The functional implication of these observations is currently unknown. Changes in cell membrane properties can be induced by the microbes themselves and by the activity of intracellular microbial effectors. It will be interesting to define whether microbial colonization–induced membrane rearrangements locally alter membrane biophysical properties and whether this is sensed to initiate appropriate cellular responses. Microbial structures such as the fungal appressorium or processes such as viral replication and movement could trigger changes in membrane biophysics and signaling. In animals, the mechanosensitive ion-permeable channel Piezo1 initiates immune responses upon sensing membrane ruffling during bacterial infection [107]. Piezo, the tonoplast-located Arabidopsis ortholog of Piezo1, is genetically required for disease resistance against TuMV [108]. As TuMV closely associates with the tonoplast, Piezo may sense tonoplast modifications during infection. Microbial colonization imposes important membrane curvatures, which in the context of symbiosis have been recently proposed to be maintained by REMORINs, proteins that act as structural components of the plasma membrane, in cooperation with actin elements [109].

Biemission probes such as Laurdan and di-4-ANEPPDHQ are commonly used to assess changes in plasma membrane biophysical properties upon contact with microbial components [97,105,106]. Recently, a microviscosity map of the plant cell was generated using a set of molecular rotor probes specific to different cellular compartments [110]. Among them, the plasma membrane-specific probe, N^+^-BODIPY, helped reveal the distinct penetration strategies of *Magnaporthe oryzae* [111] and *Phytophthora infestans* [112]. These observations encourage further exploration of the functional roles of membrane biophysics in plant-microbe interactions.

## The roles of membrane lipids

Membrane lipids can serve simultaneously as solvents and regulatory cofactors for membrane protein activity, constituting so-called functional paralipidomes [113]. Lipids can recruit proteins in specific cellular compartments [114] and can support protein lateral organization and function [34,115]. Accumulating evidence indicates a tight, yet largely mysterious, functional interplay between membrane lipids and the plant immune system. Disturbances in sphingolipid biosynthesis are commonly associated with autoimmune phenotypes and increased defense responses [116]. Specific lipid species regulate the cellular trafficking of immune components. For instance, hydroxylated sphingolipids are required for the plasma membrane accumulation of numerous immune signaling components [117], and variation in sterol accumulation correlates with defects in ligand-induced endocytosis of FLS2 [39,118]. Membrane lipids can also function as signaling molecules. Flg22 perception induces the activation of diacylglycerol kinases responsible for phosphatidic acid production, which in turn binds to, stabilizes, and promotes the activity of RBOHD [119–121]. Ceramides have similarly been shown to bind RBOHD and are proposed to regulate its activity, although the underlying mechanism remains to be identified [122]. Together, these reports show an intimate functional interrelationship between an integral membrane protein and its lipid environment, which presumably can apply to other immune and symbiotic signaling components.

Membrane lipid composition contributes to the identity of membranes throughout the cell. For instance, phospholipid signatures at the cytosolic leaflet of organelle membranes distinguish different functional compartments [114,123]. Interestingly, the membrane interfaces formed during microbial colonization exhibit lipid compositions that largely differ from those of adjacent membranes and are further compartmentalized into subdomains [124,125]. These interfaces are characterized by specific phospholipid signatures [126–130] that may promote the recruitment of specific host factors, act as signaling molecules [114], recruit cytoskeletal elements [131], and modulate, for instance, the activity of transporters [132] at the plant–microbe interface. Tomato bushy stunt virus co-opts lipid-modifying enzymes to shape the lipidic environment of its replication sites, promoting an enrichment in phospholipids required for viral replication [133,134]. The lipid composition of PD regulates their conductivity [13,135], and it remains to be determined whether this composition is modified upon infection and contributes to the cell-to-cell movement of viruses.

Membrane lipids can also function as receptors for microbial molecules. For example, NLPs are cytolytic pathogen molecules that bind to exposed sugars of the polar head of the sphingolipid glycosylinositol phosphoceramides (GIPCs) for activity in eudicotyledons [136]. Monocotyledons accumulate GIPCs with an additional sugar, which is thought to prevent NLPs from inserting into membranes [136]. Nonetheless, NLP proteins produced by monocotyledon pathogens can be cytolytic [137]. Plant resistance to these NLPs correlates with sphingolipid abundance rather than GIPC head-group complexity, suggesting a distinct sphingolipid-dependent mechanism. Another example comes from the lipopeptide surfactin, which binds to glucosylceramides and induces immune responses [97]. These examples suggest that the role of cell-surface lipids in microbial perception may be more prominent than currently appreciated.

## Conclusions and perspectives

Plant membranes host key processes involved in the perception and the transmission of microbe-induced signals, while accommodating changes in membrane topology. Apart from influencing microbial processes and affecting host protein subcellular localization, lateral organization, and activity, host membrane lipids recently emerged as receptors for microbial molecules. Plant–microbe interactions induce an intense rewiring of host membrane composition and topology. Such drastic modifications likely affect the biophysical properties of membranes and impact the signaling processes they host, but also initiate signaling on their own. These considerations open exciting prospects for future research.
